# ERP evidence on how gaze convergence affects social attention

**DOI:** 10.1038/s41598-019-44058-w

**Published:** 2019-05-20

**Authors:** Nanbo Wang, Shan Xu, Shen Zhang, Yiqi Luo, Haiyan Geng

**Affiliations:** 1 Beijing Key Laboratory of Behavior and Mental Health, School of Psychological and Cognitive Sciences, Peking University, Beijing, China; 2 Faculty of Psychology, Beijing Normal University, Beijing, China; 3 Department of Psychology, University of Wisconsin-Whitewater, Whitewater, WI USA

**Keywords:** Attention, Social behaviour, Human behaviour

## Abstract

How people process gaze cues from multiple others is an important topic but rarely studied. Our study investigated this question using an adapted gaze cueing paradigm to examine the cueing effect of multiple gazes and its neural correlates. We manipulated gaze directions from two human avatars to be either convergent, created by the two avatars simultaneously averting their gazes to the same direction, or non-convergent, when only one of the two avatars shifted its gaze. Our results showed faster reaction times and larger target-congruency effects following convergent gazes shared by the avatars, compared with the non-convergent gaze condition. These findings complement previous research to demonstrate that observing shared gazes from as few as two persons is sufficient to enhance gaze cueing. Additionally, ERP analyses revealed that (1) convergent gazes evoked both left and right hemisphere N170, while non-convergent gazes evoked N170 mainly in the hemisphere contralateral to the cueing face; (2) effects of target congruency on target-locked N1 and P3 were modulated by gaze convergence. These findings shed light on temporal features of the processing of multi-gaze cues.

## Introduction

The use of perceived gaze direction to guide visual attention is crucial to processing social information^1^. This phenomenon has been substantially investigated with dyadic paradigms in which one person interacts with another human agent or avatar^2^. Such research has demonstrated quicker responses toward an object of mutual interest, i.e., a gazed-at target, compared to other targets, even when the gaze is not predictive (the target-congruency effect, also called the gaze cueing effect)^3^. Compared to laboratory scenarios that involve a single interaction partner, interpersonal situations in the real world are often more complex. For example, at a party people may encounter more than one individual at a time and exchange gazes with multiple persons. Sometimes the crowd may gaze at the same direction, and we refer to this kind of group gazes as convergent gazes; at other times, people may not all look toward the same direction, and we refer to such group gazes as non-convergent gazes. How do social cues, specifically convergent versus non-convergent gazes, affect gaze cueing of attention?

So far there has been limited research examining the effects of social gaze in a group setting^4–8^. Some studies found that multi-gaze cues can impact affective evaluation of objects. Specifically, objects looked at by multiple faces were liked more than objects looked away from, but this difference was absent when the object was looked at or away by a single face^9^. Such results suggest that multiple gazes may be perceived as more reliable and trustworthy sources about the valence qualities of objects, compared with a single gaze^9,10^. In line with our research interest, gaze information from multiple people has been found to affect individuals’ visual attention in field studies. Milgram *et al*.^4^ and Gallup *et al*.^5^ had groups of confederates stare up at a building in the street, and measured the likelihood of passing pedestrians adopting this behavior. The results showed that the probability of a pedestrian following the group’s action increased as a function of increased number of confederates. Findings like these suggest that more convergent gazes from the group lead to stronger gaze following effect. Similar patterns were found in laboratory settings as well. Sun *et al*.^6^ and Capozzi *et al*.^7^ presented to participants a group of human avatars with different gaze orientations, and then a target appeared left or right to the avatars. When not all the avatars gazed at the same direction, the participants followed the direction of the gazes shared by the majority of avatars, and as the ratio of avatars that gazed at the same direction gradually increased, the induced gaze cueing of attention also increased (the majority effect). The strongest cueing effect occurred when all avatars looked in the same direction. Although the researchers emphasized a positive association between the number of gazes shared and subsequent gaze cueing of attention, for us, these findings can also be understood as convergent gazes (when all avatars had the same gaze direction) overall having a stronger effect in cueing attention, compared with non-convergent gazes (when not all the avatars had the same gaze direction).

However, the underlying neural mechanisms in the processing of convergent and non-convergent gazes have not been explored. In addition, the non-convergent gaze condition in previous studies often included gazes congruent and incongruent to the target location in the same trial^6,7^ (while some of the avatars were looking towards the target, other avatars were looking in the opposite direction of the target), which may create difficulty in dissociating neural activities corresponding to congruent and incongruent gaze cues in neuroimaging research. Therefore, we adopted a modified paradigm and recorded event-related electrical potentials (ERPs) to examine the potential distinction between the processing of convergent and non-convergent gazes at the neural level, in addition to test whether previous behavioral findings regarding these two types of gazes can be replicated. Specifically, we adopted a triadic context, the smallest unit of a crowd scenario, with the presentation of two avatars’ faces to contrast convergent and non-convergent gaze processing. The *convergent gaze condition* was established with the gazes of the two faces simultaneously shifting towards the same direction, either directing towards a target (the congruent trials) or away from it (the incongruent trials), whereas in the *non-convergent gaze condition*, only one of the two faces shifted its gaze. Participants were asked to detect the target which might randomly appear on either the left or the right side of the faces (independent of the gaze shift).

We expected that convergent and non-convergent gazes have different effects at both the behavioral and the neural levels. At the behavioral level, we expected to find that the convergence between gaze cues increases the gaze cueing of attention, consistent with previous research^6,7^. At the neural level, if convergent and non-convergent gazes are perceived differently, the distinction may take place in the early processing of gaze stimuli, even before the onset of the target, and such difference may be observed in the cue-locked ERP analysis related to gaze and face processing. In the present study, for cue-locked analysis we focused on N170, a component sensitive to the detection of face and gaze stimuli^11–15^. Stimuli such as averted gazes and eye shifts trigger larger N170 compared with straight gazes^16,17^. If gaze convergence alters perception of the faces and gaze cues, one would expect a dissociation between N170 elicited by gazes that converge and those do not.

It is also possible that the two types of gaze cues affect the processing of the target. Therefore we compared ERP components time-locked to the onset of the target, specifically N1 and P3, between the convergent and the non-convergent gaze conditions (target-locked analysis). N1 is an early visual-evoked ERP component, and is thought to index perceptual processing in the brain^18–20^. In a typical spatial cueing paradigm, N1 is found to be larger for a congruent target that appears in the same direction of the gaze cue (compared with an incongruent target), and such difference is believed to reflect enhanced perceptual processing of the congruent target at its onset^21,22^. P3, on the other hand, is a late ERP component related to stimulus evaluation or categorization^23,24^. Improbable yet task-relevant events tend to elicit larger P3 comparing to more probably or task-unrelated events^25,26^. This component has also been demonstrated sensitive to target congruency in gaze cueing paradigms: congruent targets consistently produce smaller P3 than incongruent targets^1,21,27–29^. In the present study, we expected target-congruency effects similar to previous research on N1 and P3 components. Moreover, we expected that such effects may be modulated by gaze convergence. Specifically, convergent gazes may produce stronger attention shift to the gazed direction compared with non-convergent gazes, consequently augment perception of the target appearing in the gazed-at location (manifested as a larger N1), and result in a larger target-congruency effect. In addition, if convergent gazes are considered more reliable^9^, they may raise participants’ expectation for the target to appear in the gazed-at location (manifested as a smaller P3), but not in the non-gazed-at location. Again we predicted a larger target-congruency effect following convergent gazes compared to non-convergent gazes.

In summary, the present study investigated how convergent and non-convergent gazes from two human avatars affect social attention with a modified gaze cueing paradigm. We hypothesized that convergent gaze cues would induce a larger target-congruency effect comparing to non-convergent gaze cues. In addition, we explored whether convergent and non-convergent gazes would be processed differently (by analyzing cue-locked N170), and whether such distinctive processing of gaze cues, if exists, has different impacts on the early target perception (by analyzing target-locked N1), as well as the late decisional processing of target stimuli (by analyzing target-locked P3).

## Method

### Participants

The sample size of our study was determined via *a priori* power analysis using G*Power 3 (Faul, Erdfelder, Buchner, & Lang, 2009). Considering the observed effect size in research of similar paradigm^6^, we estimated the effect size to be large. Given a large effect size (partial η^2^ = 0.14), a power of 0.90, and an alpha level of 0.05, the power analysis ultimately yielded an estimated sample size of 20. Data collection was terminated at the end of the day when desired number of eligible participants was achieved.

As a result, we recruited a total of 23 eligible participants with normal or corrected-to-normal vision from Peking University (10 males and 13 females, aged between 18 to 26 years, *Mean* = 21.62 years, *SD* = 2.47 years), after excluding two participants who did not finish the experiment due to errors in EEG recordings, and another two whose error rate in behavioral responses were high (out of two standard deviations of the mean)^30^.

### Materials

*Face stimuli*. Two Asian faces with neutral facial expression were generated by FaceGen Modeller 3.4.1 (Copyright © 2009, singular Inversions Inc.). Both faces subtended 3.3° × 3.3°, showing the region between the top of the heads and the jaws, symmetrical by the vertical line across their noses. The two faces were simultaneously presented in the upper left and upper right visual fields, the inner and the outer contours of each face were 1.55° and 4.85° left/right to the central of the screen, respectively, and the upper and the lower contours were 2.65° above and 0.65° below.

At the beginning of each critical trial, these two faces were presented on the screen, facing 10° inward toward each other and gazing straight ahead to imitate an interactive situation (the baseline stimuli). The faces would then shift their gazes to the left or to the right by 45° relative to the head direction, or remain still in the trial, to create different gaze cue conditions (see Procedure below). The face stimuli in the catch trials were presented the same way, except that their eyes were closed throughout the trial.

#### Fixation

A white fixation cross (0.5° × 0.5°) was presented 1° above the center of the screen throughout each trial.

#### Target

The target was a red dot (0.5° radius) presented 6.3° to the left/right and 2.9° below the center of the screen.

### Procedure

The experiment was conducted using MATLAB 7 with Psychtoolbox 3. The materials were displayed on a 17-in. View sonic Professional Series P97f+ (1024 × 768 at 100 Hz) against a black background. Participants’ heads were supported by a chin rest at a viewing distance of 60 cm from the computer screen.

Participants individually took part in the study. They first completed a practice block of 27 trials to ensure that the participants understood the instructions. Then each participant completed 10 formal blocks of trials, with 72 critical trials and 9 catch trials in each formal block. Therefore each participant went through a total of 720 critical trials. Among the 72 critical trials in a single block, 48 trials showed non-convergent gazes, where only one face shifted its gaze (the non-convergent gaze condition), including 24 trials where the face in the left visual field shifted its gaze (the non-convergent LVF trials) and 24 trials where the face in the right visual field shifted its gaze (the non-convergent RVF trials). In the remaining 24 critical trials, both faces shifted gazes simultaneously to the same direction, either left or right (the convergent gaze condition). Similarly, the target randomly appeared on the gazed-at side for half of the trials (the congruent target condition), and on the opposite side for the other half of the trials (the incongruent target condition). The order of trials was randomized in each block, and trials from a same condition would not appear more than three times in succession.

**Figure 1:**
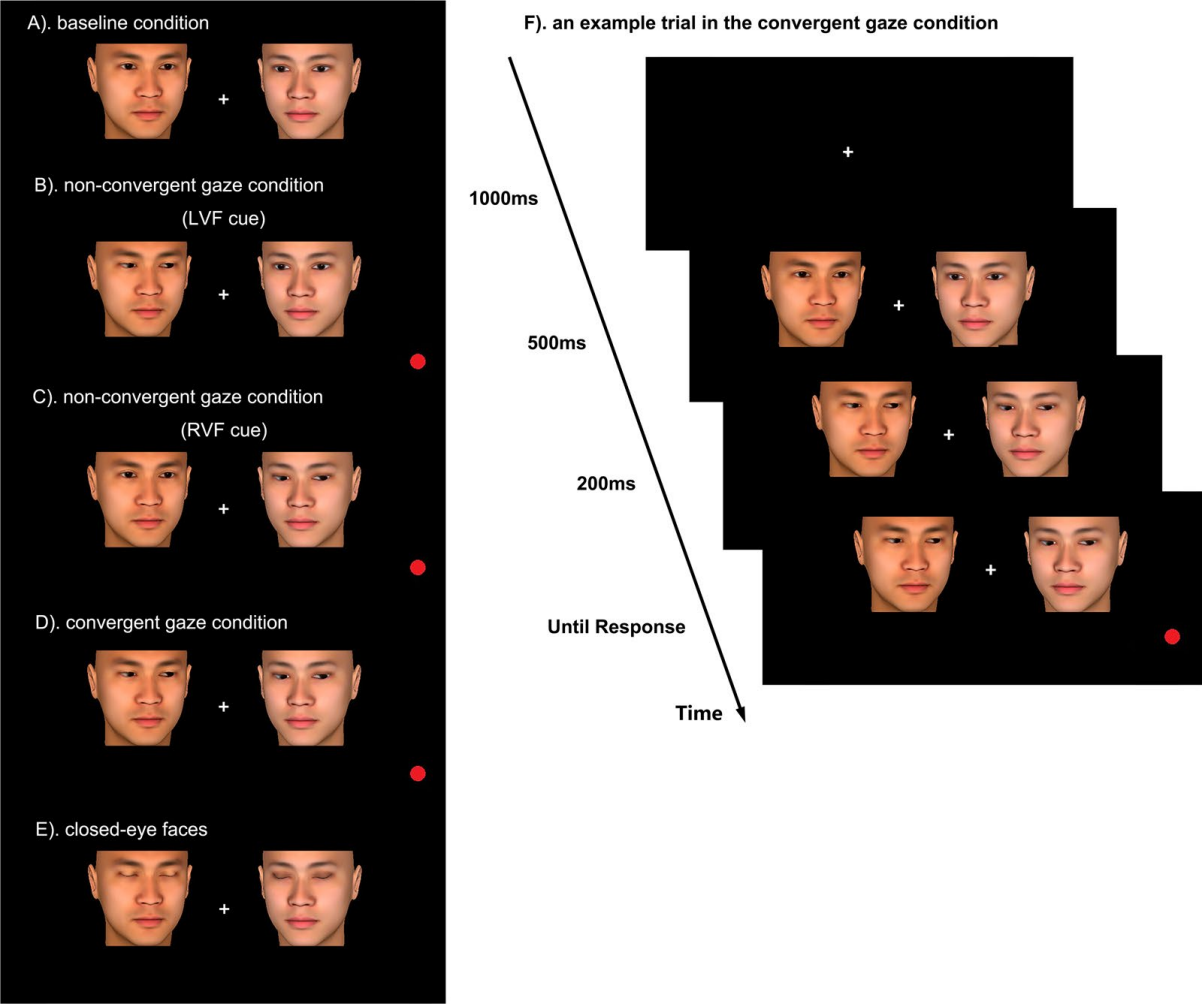
Examples of face stimuli (**A**–**E**) and a critical trial (**F**). (**A**) The baseline condition. (**B**) The non-convergent LVF gaze condition, in which the left face shifts its gaze (in this example, to the right) while the right face maintains its initial gaze. (**C**) The non-convergent RVF gaze condition, in which the right face shifts its gaze (also to the right in this example) while the left face maintains its initial gaze. (**D**) The convergent gaze condition, in which the two faces shift their gazes to the same direction (left in this example). (**E**) The closed-eye faces in the catch trials. (**F**) An example trial in the convergent gaze condition with a congruent target. In this example, both gazes shift to the right direction, and the target appears on the right side of the screen.

Each critical trial started with a 1000 ms presentation of the fixation. Then the participants viewed the baseline stimuli for 500 ms (see Fig. 1). After that, one of the two faces (the non-convergent gaze condition), or both of them (the convergent gaze condition), shifted its gaze/their gazes either to the left or to the right. After a 200 ms latency, the target appeared either left or right to the faces and remained until response. Then the next trial began after a 1500~2500 ms blank screen interval. Participants were informed beforehand that gaze direction was irrelevant to the target’s location. They were asked to ignore the gaze shift and report as quickly as possible the position of the target by pressing the Left Arrow or the Right Arrow key.

The catch trials were included to keep the participants’ attention on the presented face stimuli. These trials presented faces with closed eyes but no target. Participants were told to press the ‘SPACE’ key as quickly as possible whenever they saw any closed-eye face.

The datasets generated during and/or analysed during the current study are available from the corresponding author on reasonable request.

### ERP recordings and data analysis

The EEG data were recorded and processed using a 64 Brian Products channel device (the International 10–20 System). The horizontal and vertical electrooculography (EOG) were recorded by placing two bipolar electrodes at the outer canthi of the left eye (HEOG) and at the supraorbit of the right eye (VEOG). The online reference was positioned on the tip of the nose, and then re-referenced offline to the average of left and right mastoids. All electrode impedances were kept below 10 kΩ. The EEG data were amplified at a band pass of 0.01–100 Hz, and digitized at 500 Hz. Trials with eye movements, blinks (using independent component analysis, ICA) or other artifacts (a voltage exceeding ±70 μV at any electrode) were rejected offline and were not included in the analysis.

For cue-locked analyses, with N170 as the ERP component of interest, the EEG data were segmented into 350 ms epochs (from 100 ms before to 250 ms after cue onset). The baseline was corrected against the mean voltage during the 100 ms pre-cue period (−100–0 ms). N170 (peaking at approximately 180 ms) was quantified as the mean amplitude within a post-cue time window of 160–210 ms at temporal-occipital electrode sites TP7/TP8, CP5/CP6, P7/P8 and PO7/PO8. We took the average of TP7, CP5, P7 and PO7 as the left hemisphere amplitude, the average of TP8, CP6, P8 and PO8 as the right hemisphere amplitude.

For target-locked analyses, with N1 and P3 as the ERP components of interest, the EEG data were segmented into 700 ms epochs (from 100 ms before to 600 ms after target onset), and the corrected-baseline was the mean voltage during the 100 ms after target onset (0–100 ms). The mean amplitude of N1 was measured at central anterior electrode sites FC1, FCZ and FC2 in a time window of 150–210 ms from target onset. The mean amplitude of P3 was measured at centroparietal electrode sites CPZ, CP1, CP2 in a time window from 350 ms to 450 ms after target onset.

In cue-locked analysis, given that the representation of the gaze presented in each visual field was lateralized^31^, to dissociate the neural responses to each face, we included hemisphere (left vs right) and gaze cue (non-convergent LVF gazes vs. non-convergent RVF gazes vs. convergent gazes) as the within-participant factors in ANOVA. In the target-locked analysis, we included gaze cue (convergent gazes vs. non-convergent gazes) and cue-target congruency (congruent target vs. incongruent target) as the within-participant factors. Bonferroni correction was applied to all post hoc pairwise comparisons in both behavior and ERP analyses reported in this paper.

### Ethics statement

This study was approved by the Ethics Review Committee of Peking University. Written informed consent was obtained from all participants before the experiment in accordance with the Declaration of Helsinki.

## Results

### Behavioral results

Participants’ average accuracy on recognizing the closed-eye faces in the catch trials was 98.89%, and their average accuracy of reporting the position of the target in the formal trials was 97.36%. Only correct responses with reaction times (RTs) between 200 ms to 1200 ms were subjected to analyses.

### Reaction time

**Figure 2:**
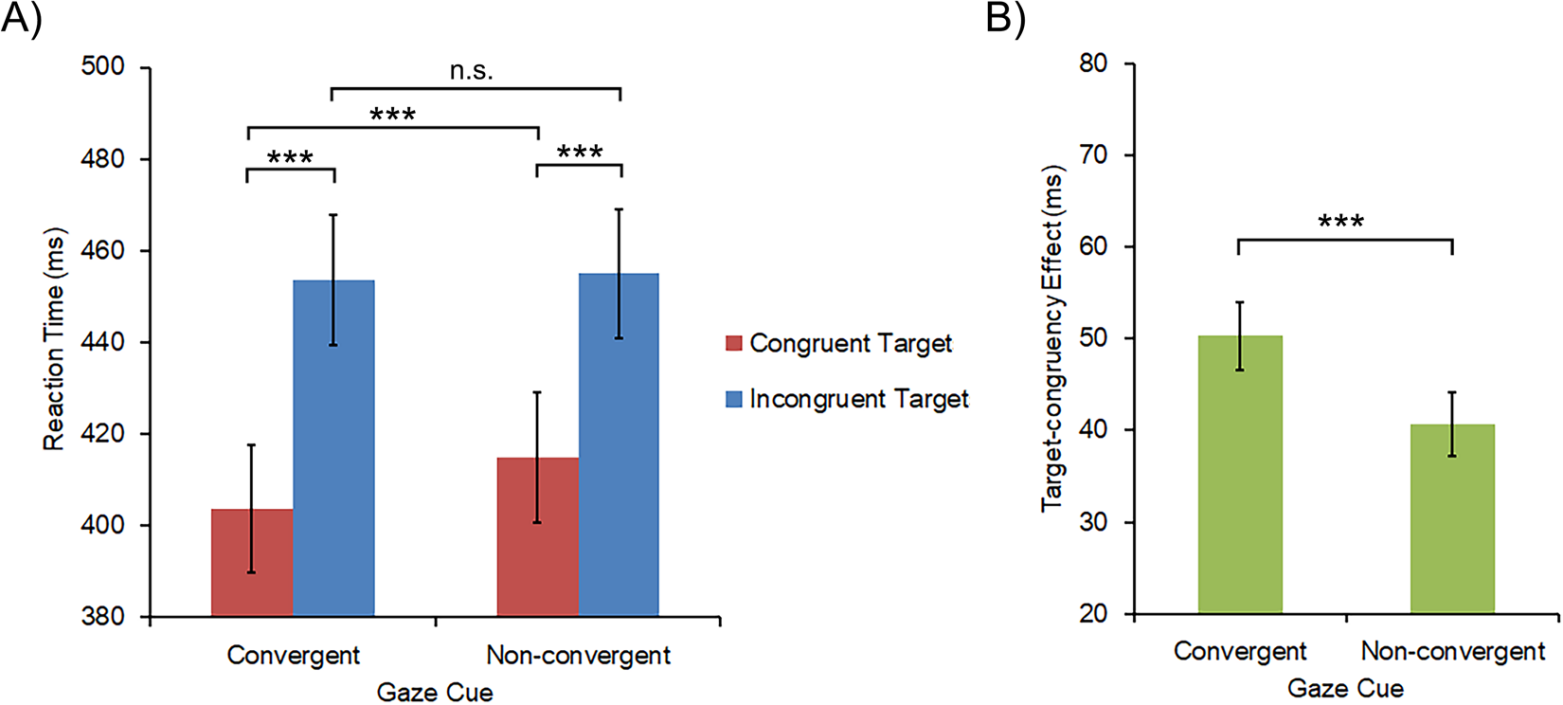
(**A**) Participants’ mean reaction times in trials with a congruent target and an incongruent target following the convergent and the non-convergent gaze cues. (**B**) Participants’ target-congruency effects in the convergent and the non-convergent gaze conditions. The error bars represent one standard error. The asterisks indicate significant pairwise comparisons, ***p < 0.001.

We conducted a 2 × 2 repeated-measure ANOVA on the RTs, with Gaze Cue (convergent gazes vs. non-convergent gazes) and Cue-target Congruency (congruent target vs. incongruent target) as within-participant factors. Significant main effects were found for both Gaze Cue and Cue-target Congruency. Overall participants responded faster following convergent gazes compared to non-convergent gazes, *F* (1, 22) = 18.798, *p* < 0.001, *η*^2^ = 0.461. Likewise, faster responses were found in the congruent than in the incongruent target condition, *F* (1, 22) = 178.269, *p* < 0.001, *η*^2^ = 0.890. These main effects were qualified by the significant interaction between Gaze Cue and Cue-target Congruency, *F* (1, 22) = 36.946, *p* < 0.001, *η*^2^ = 0.627. Post hoc pairwise comparisons revealed that faster responses were consistently found for congruent targets than incongruent targets (*ps* < 0.001) in each gaze cue condition. Meanwhile, faster responses following convergent gazes than non-convergent gazes were only found for congruent targets (*p* < 0.001), but not for incongruent targets (*p* = 0.396, see Fig. 2A and Table 1).

**Table 1:** Mean (ms) and stand error for participants’ reaction times.

	Congruent target	Incongruent target	Overall
Convergent gazes	403.68 (13.93)	453.61 (14.14)	428.64 (13.92)
Nonconvergent gazes	414.89 (14.17)	455.04 (14.04)	434.96 (14.01)
Overall	409.28 (14.02)	454.33 (14.06)	

To further understand the above interaction, we calculated the target-congruency effect by subtracting RTs of congruent-target trials from that of incongruent-target trials (target-congruency effect = RT_inc_ − RT_con_) and compared that between the two gaze cue conditions with a paired-sample *t*-test. As hypothesized, the results showed a significantly larger target-congruency effect in the convergent gaze condition (*M* = 50.31, *SE* = 3.75) than in the non-convergent gaze condition (*M* = 40.70, *SE* = 3.45), *t* (22) = −5.853, *p* < 0.001 (Fig. 2B).

### ERP results

#### Cue-locked N170

**Figure 3:**
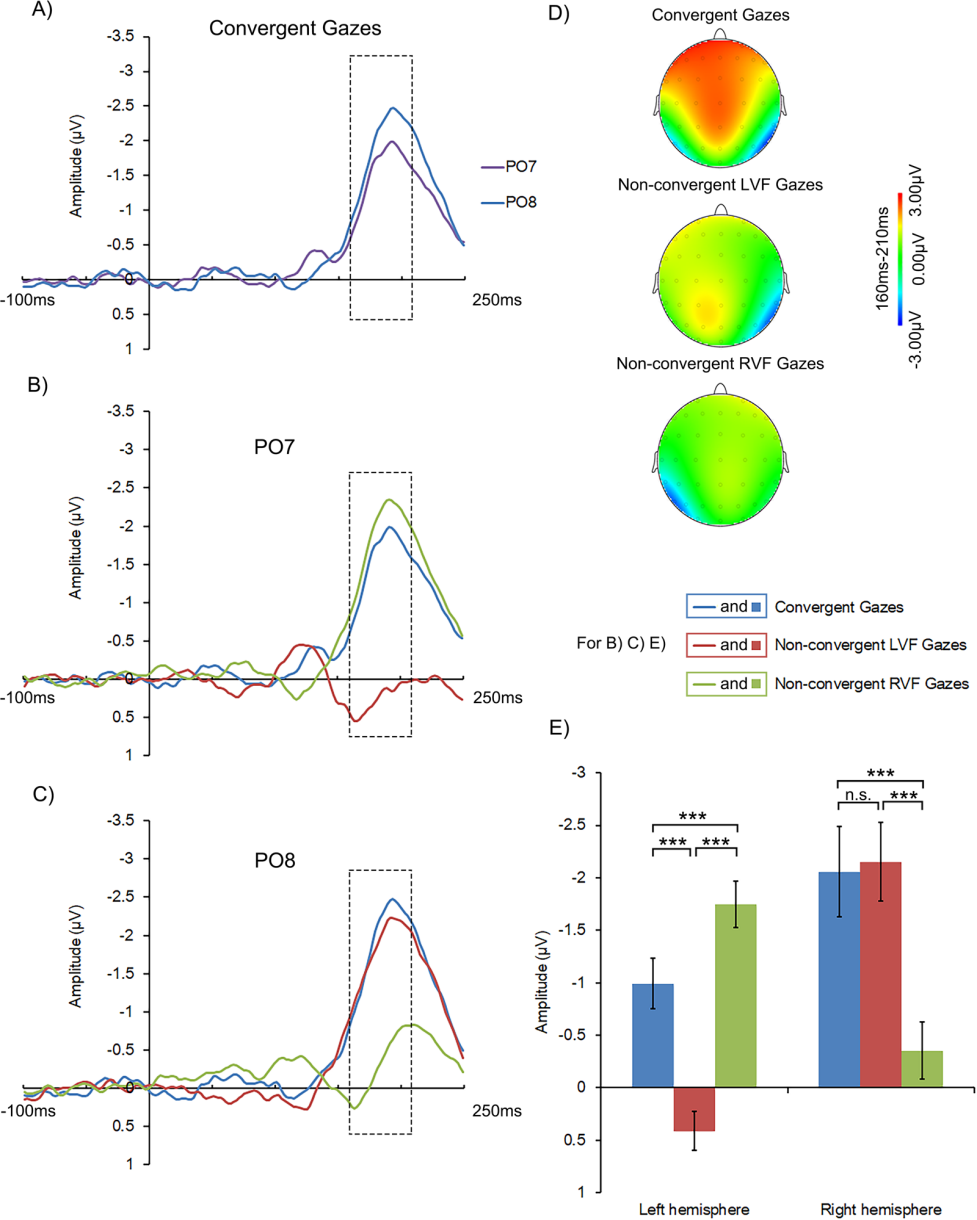
(**A**) N170 waveforms for convergent gazes at PO7 and PO8. (**B**,**C**) N170 waveforms for convergent gazes, non-convergent LVF gazes and non-convergent RVF gazes at PO7 and PO8 respectively. The frames indicate the time window from which the EEG amplitudes were averaged as an estimation of N170 amplitude. (**D**) N170 topographical maps in the trials with convergent gazes, non-convergent LVF gazes and non-convergent RVF gazes. (**E**) The mean amplitudes of N170 in the left and the right hemispheres for the convergent, the non-convergent LVF and the non-convergent RVF gazes. In (**B**,**C**,**E**), the blue color denotes the convergent gaze condition, the red the non-convergent LVF gaze condition and the green the non-convergent RVF gaze condition. The error bars represent one standard error. The asterisks indicate significant pairwise comparisons, ***p < 0.001.

We conducted a 3 × 2 repeated-measure ANOVA on N170 with Gaze Cue (convergent gazes vs. non-convergent LVF gazes vs. non-convergent RVF gazes) and Hemisphere (left hemisphere vs. right hemisphere) as within-participant factors, in order to differentiate the ipsilateral and the contralateral hemispheres N170 elicited by the gaze cues. There was a significant main effect of Gaze Cue, *F* (2, 44) = 13.627, *p* < 0.001, *η*^2^ = 0.382, with larger N170 amplitude in the convergent gaze condition than in the non-convergent LVF gaze (*p* < 0.001) or non-convergent RVF gaze condition (*p* = 0.010). No significant difference was found between the non-convergent LVF and RVF gaze trials. The main effect of Hemisphere was also significant, *F* (1, 22) = 4.426, *p* = 0.047, *η*^2^ = 0.167, with larger N170 in the right hemisphere than in the left hemisphere. These main effects were qualified by the significant interaction between Gaze Cue and Hemisphere, *F* (2, 44) = 106.648, *p* < 0.001, *η* ^2^ = 0.829 (Fig. 3E and Table 2, see also Fig. 3D for the N170 topographical map under each condition). Specifically, post hoc comparisons showed that following convergent gazes, larger N170 was found in the right hemisphere compared to that in the left hemisphere, *p* = 0.028 (see Fig. 3A for N170 waveforms from representative electrodes in the left and the right hemispheres). Although the same lateralization was found for non-convergent LVF gazes, for non-convergent RVF gazes it was opposite: larger N170 in the left hemisphere than in the right hemisphere, *ps* < 0.001. In fact, convergent gazes evoked N170 in both the left and the right hemispheres, but non-convergent gazes evoked N170 mainly in the hemisphere contralateral to the cueing face (LVF gaze shift mainly evoked N170 in the right hemisphere, and RVF gaze shift mainly evoked N170 in the left hemisphere), as can be seen from the N170 waveforms and the topographical maps. Meanwhile, in the left hemisphere, N170 elicited by the non-convergent RVF gazes and the convergent gazes were both larger than that elicited by the non-convergent LVF gazes (*ps* < 0.001), while the N170 elicited by the non-convergent RVF gazes was the largest (*p* < 0.001, see Fig. 3B for N170 waveform from a representative electrode in the left hemisphere). In the right hemisphere, similar N170 were elicited by the non-convergent LVF gazes and the convergent gazes (*p* > 1), and both were larger than that elicited by the non-convergent RVF gazes (*ps* < 0.001, see Fig. 3C for N170 waveforms from a representative electrode in the right hemisphere).

**Table 2:** Mean (μV) and stand error for N170 amplitude.

	Hemisphere		Overall
	Left	Right	
Convergent gaze	−0.99 (0.24)	−2.06 (0.43)	−1.52 (0.27)
Non-convergent LVF gaze	0.41 (0.19)	−2.15 (0.38)	−0.87 (0.23)
Non-convergent RVF gaze	−1.75 (0.22)	−0.35 (0.27)	−1.05 (0.19)
Overall	−0.78 (0.19)	−1.52 (0.34)	

#### Target-locked analyses

We conducted separate 2 × 2 repeated-measure ANOVAs on the mean amplitudes of N1 and P3, with Gaze Cue (convergent gazes vs. non-convergent gazes) and Cue-target Congruency (congruent target vs. incongruent target) as within-participant factors in each ANOVA.

#### N1

**Figure 4:**
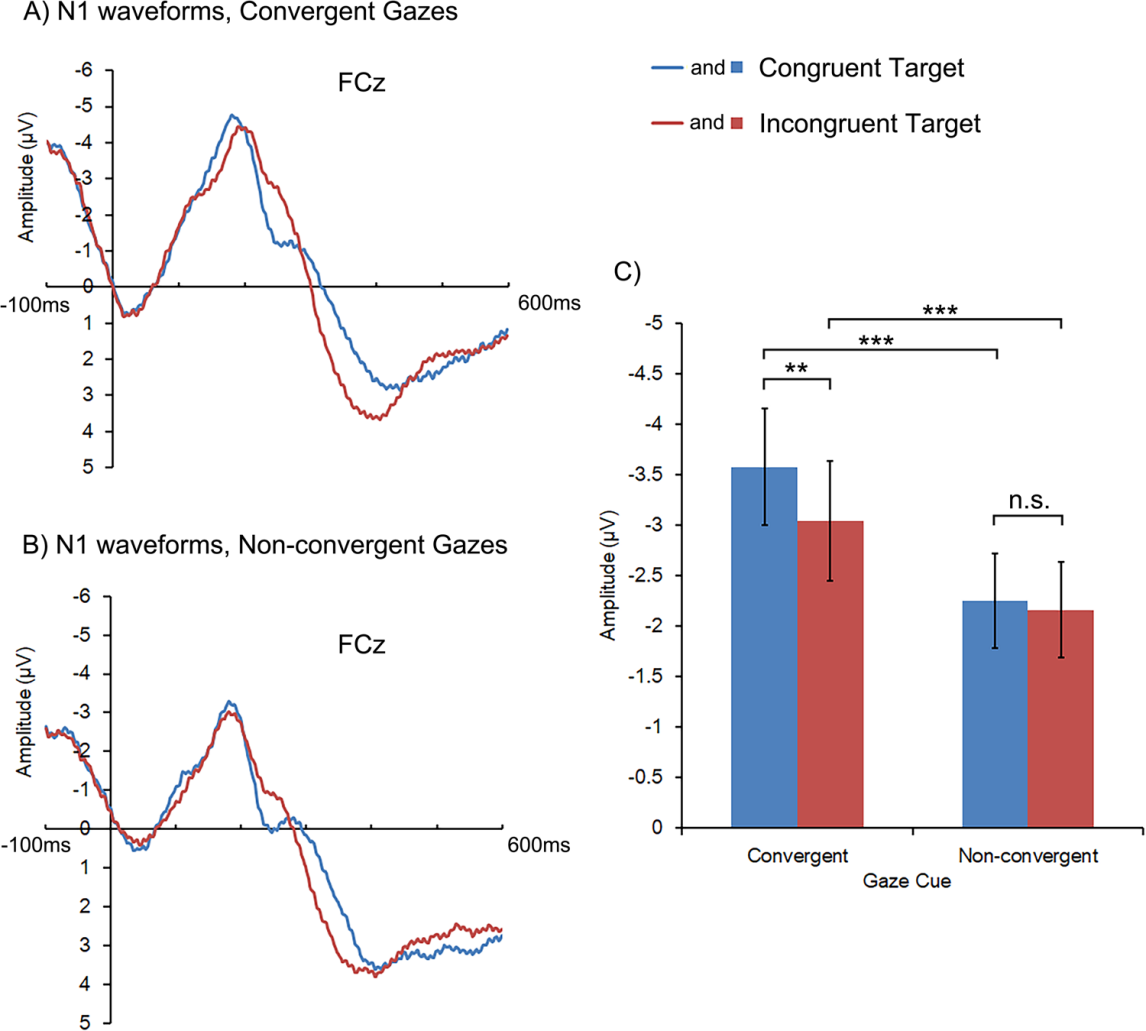
Target-locked N1 waveforms (at FCz) and the mean amplitudes for congruent and incongruent targets following convergent and non-convergent gazes. The blue color denotes the congruent target condition, and the red the incongruent target condition. The frames indicate the time window from which the EEG amplitudes were averaged as an estimation of N1. The error bars represent one standard error. The asterisks indicate significant pairwise comparisons, **p < 0.01, ***p < 0.001.

Both main effects were significant. In general, the mean amplitude of N1 was larger in the convergent gaze condition than in the non-convergent gaze condition, *F* (1, 22) = 41.191, *p* < 0.001, *η*^2^ = 0.652, as well as in the congruent target trials than in the incongruent target trials, *F* (1, 22) = 6.158, *p* = 0.021, *η*^2^ = 0.219. These main effects were further qualified by the significant interaction between Gaze Cue and Cue-target Congruency, *F* (1, 22) = 5.681, *p* = 0.026, *η*^2^ = 0.205. Specifically, post hoc comparisons showed that larger N1 in congruent than incongruent target trials was only found in the convergent gaze condition (*p* = 0.002, Fig. 4C and Table 3, see also Fig. 4A,B for the waveforms of N1 from a representative electrode site), not in the non-convergent gaze condition (*p* = 0.570). Additionally, larger N1 following convergent than non-convergent gazes was found for both congruent and incongruent targets (*ps* < 0.001).

**Table 3:** Mean (μV) and stand error for N1 and P3 amplitude.

	N1		Overall	P3		Overall
	Congruent target	Incongruent target		Congruent target	Incongruent target	
Convergent gazes	−3.58 (0.58)	−3.04 (0.60)	−3.31 (0.58)	3.45 (0.95)	5.35 (1.04)	4.40 (0.98)
Nonconvergent gazes	−2.25 (0.47)	−2.16 (0.47)	−2.21 (0.47)	4.35 (0.95)	5.33 (0.95)	4.84 (0.94)
Overall	−2.91 (0.52)	−2.60 (0.53)		3.90 (0.95)	5.34 (0.99)	

#### P3

**Figure 5:**
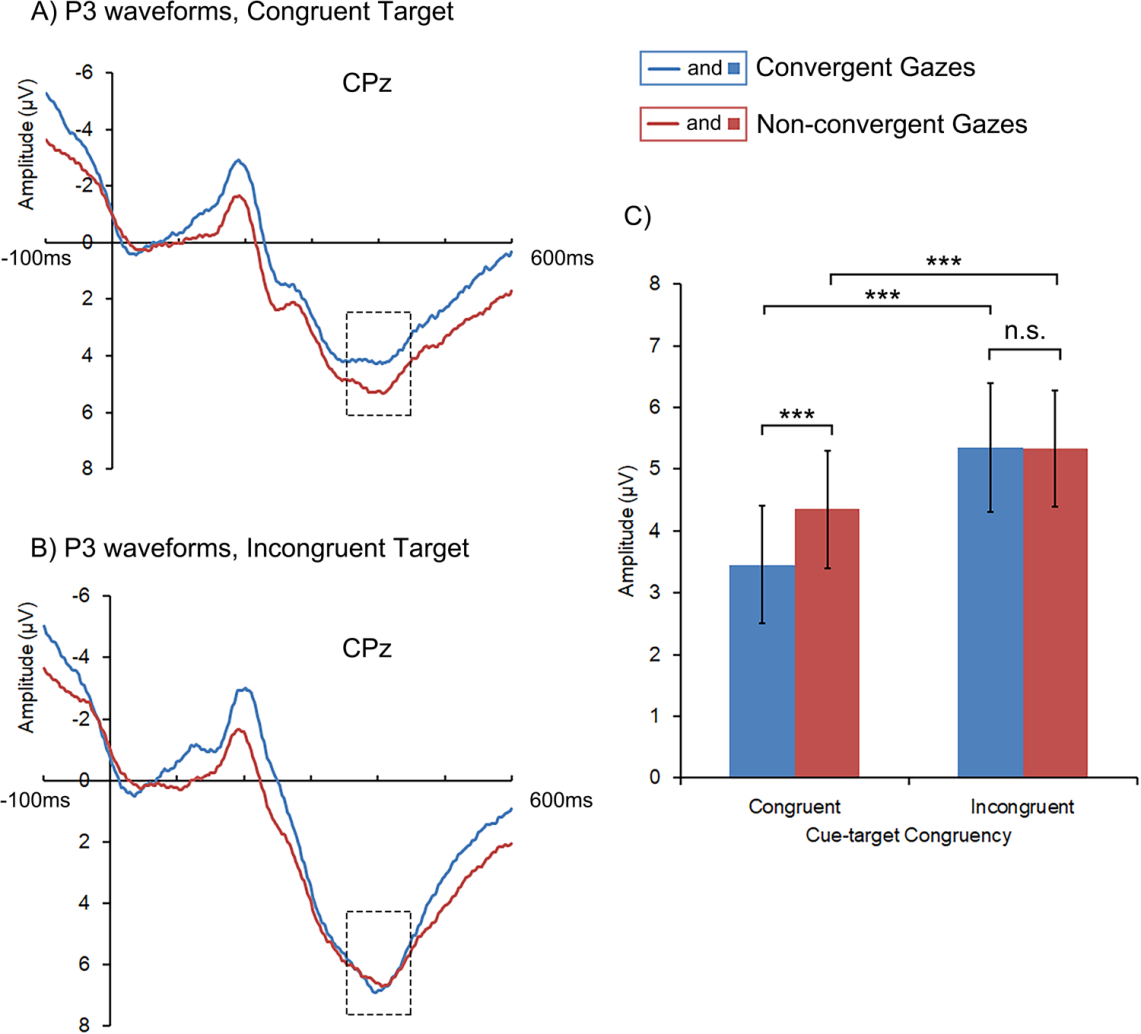
Target-locked P3 waveforms (at CPz) and the mean amplitudes for congruent and incongruent targets following convergent and non-convergent gazes. The blue color denotes the convergent gaze condition, and the red the non-convergent gaze condition. The frames indicate the time window from which the EEG amplitudes were averaged as an estimation of P3. The error bars represent one standard error. The asterisks indicate significant pairwise comparisons, ***p < 0.001.

Similar to the results on N1, both main effects of Gaze Cue and Cue-target Congruency were significant. Smaller P3 was found for congruent than incongruent targets, *F* (1, 22) = 26.364, *p* < 0.001, *η*^2^ = 0.545, as well as following convergent gazes than non-convergent gazes, *F* (1, 22) = 9.151, *p* = 0.006, *η*^2^ = 0.294. The interaction between Gaze Cue and Cue-target Congruency was significant, *F* (1, 22) = 8.778, *p* = 0.007, *η*^2^ = 0.285. Post hoc pairwise comparisons revealed smaller P3s in detecting congruent targets compared with detecting incongruent targets for both gaze-cue conditions (*ps* < 0.001). As expected, smaller P3 following convergent gazes than non-convergent gazes was observed for the congruent targets (*p* < 0.001, Fig. 5C and Table 3, see also Fig. 5A,B for the waveforms of P3 from a representative electrode site), but this effect was not found for the incongruent targets.

## Discussion

Emerging research examining the perception of shared gazes from multiple people has demonstrated a majority effect on gaze cueing of attention^6,7^, but the neural mechanism underlying the distinction between convergent and non-convergent gazes in affecting gaze cueing remained unclear. The present study explored this question by utilizing a modified gaze-cueing paradigm to compare convergent gazes created by two human faces shifting their gazes towards the same direction and non-convergent gazes in which only one of the two faces shifts its gaze. At both the behavioral and the neural levels, we found a significant target-congruency effect in that participants reacted faster to the congruent targets compared with the incongruent targets, accompanied by larger N1 and smaller P3 amplitudes, and these findings were consistent with previous research^3,21,27,29^.

One goal of our research is to examine possible dissociation between convergent and non-convergent gaze cues. As expected, we found that target-congruency effects were modulated by gaze convergence. At the behavior level, the participants showed larger target-congruency effects and faster responses following convergent gazes compared with non-convergent gazes, probably because multi-gaze cues in the same direction may increase the strength of cueing or the perceived reliability of these gazes, and thus facilitate gaze cueing^6^. In addition, faster responses following convergent gazes (versus non-convergent gazes) were only found for congruent targets but not for incongruent targets, which was consistent with previous research showing incongruent trials less susceptible to top-down manipulations in a gaze cueing task^32^.

Although previous studies had examined the effect of gaze convergence^6,7^, their “non-convergent” gaze condition consisted of gazes both congruent and incongruent to the target in the same trial, which might underestimate the effect of attention cueing in this condition. Therefore including cue-target congruency as a factor independent of gaze convergence in our study allows a clearer understanding of the cueing effect of multiple gazes. Meanwhile, we extended previous research paradigm and simulated a common scenario of non-convergent gazes in daily life, in which some people gaze toward a given stimulus while others may not pay attention.

More importantly, we found consistent neural evidence for the distinctive effects of the two types of multi-person gaze cues. First, convergent and non-convergent gazes were initially processed differently, as evidenced by dissociated N170s. Specifically, convergent gazes evoked N170 in both the left and the right hemispheres, but non-convergent gazes evoked N170 mainly in the hemisphere contralateral to the cueing face, e.g., the gaze shift presented in the left visual field (non-convergent LVF gazes) evoked much larger N170 in the right hemisphere but no N170 in the left hemisphere. A possible reason for such dissociation is the lateralized processing of gaze shift^31^, i.e. the gaze cues from each visual field were processed preferably in the contralateral cerebral hemisphere. Bihemispheric N170s in the convergent gaze condition suggest that gaze shifts in both visual fields were simultaneously processed at this stage, resulting in bilateral N170s of comparable amplitude. In contrast, in the non-convergent gaze condition, gaze shift occurred only in one visual field, which elicited a larger N170 comparing to the static gaze in the other visual field in their corresponding contralateral hemispheres, leading to lateralized N170^31^. These results, taken together, indicated that different gaze cues, regardless of convergent or not, were efficaciously processed.

Second, we found neural evidence that convergent gazes influenced the subsequent target processing, including strengthening visual perception and modulating the expectation of the target stimuli, as evidenced by larger N1 and smaller P3. The larger N1 amplitude was found for the congruent targets in relative to the incongruent targets, consistent with the case of single-gaze cueing paradigms^21,22^. But this congruency effect could only be observed following convergent gazes. This may be because convergent gazes had particularly strengthened the early processing of the congruent targets. Non-convergent gazes, in contrast, contained inconsistent gaze information which possibly needed reconciliation to guide attention. Such a process might require more resources, therefore impeded the early processing of the targets, leading to lower N1 amplitude following non-convergent gazes. Moreover, smaller P3 was found following the convergent gazes comparing to the non-convergent gazes for the congruent targets; but such gaze convergence effect was absent for the incongruent targets^25,26^. These results suggest that participants seemed to expect the target to be more likely to appear at the gazed-at location following the presence of convergent than non-convergent gazes. This may again be because convergent gazes were perceived as more reliable in indicating the target’s position than non-convergent gazes^6^, and they selectively enhanced participants’ expectation that the target would appear in the gazed-at location^25,26^, whereas the expectation for target in the non-gazed-at location was not affected.

Finally, our results also shed light on the time course of integration of multi-gaze cues. Since EEG is a continuous record of brain activity with high temporal resolution, the temporal sequence of ERP components can indicate the time course of related cognitive processes^33–37^. For example, face invoked N170 and target invoked N1 and P3 may respectively correspond to face processing, and early stage as well as late stage of target processing^38–40^. In our research, we were able to infer the time course of gaze integration by examining at which ERP component the gaze convergence effect started to emerge. Note there was inconsistency between behavioral results and N170 findings: although participants reacted significantly faster following convergent gazes than non-convergent gazes, the amplitude of N170 in each hemisphere in the convergent gaze condition did not significantly surpass their counterparts in the non-convergent gaze condition. Based on these evidence, we speculate that the integration of multiple gaze cues emerges at a stage later than N170. When multiple gazes from different visual fields are represented, they are first processed separately in the contralateral hemisphere where N170 is evoked^31^. Consistently, the neural activities induced by gaze cues from a single visual field were comparable between the convergent and non-convergent gaze conditions (see Fig. 3). However, differences between the convergent and the non-convergent gaze conditions were evident in the processing of the subsequent target, as shown in N1 and P3 components in the present study (see Figs 4 and 5). Such results may indicate that the integration of multi-gaze cues had been achieved at these time points, and further reflected in participants’ behavioral responses. Taken together, our findings suggest that the inter-hemispheric integration of gaze information may only start to affect gaze cueing in later stage of gaze processing (at least after the time point of N170) or even after the onset of targets.

Interestingly, although the behavioral target-congruency effect following convergent gazes was mirrored in both N1 and P3 amplitudes, following non-convergent gazes the counterpart effect was only found in P3, not in N1. The results suggest that the target-congruency effect in the convergent gaze condition may be driven by the impact of gaze cues both on the early and the late processing of target, while the target-congruency effect following non-convergent gazes may be predominantly driven by the impact of gaze cues on the late processing of target. As discussed above, this is probably because convergent gazes enhanced the perception of the directional information carried by each face as well as the expectation of the congruent target, therefore affected both the early and late processing of target and produced the target-congruency effect on both N1^18–20^ and P3^25,26^. In the non-convergent gaze condition, however, gaze inconsistency from the two faces need to be resolved first to guide attention, therefore the target-congruency effect only emerged later and was reflected in the decisional or evaluating stage of target processing (in P3^25,26^, but not N1). Future research should verify these speculations on the temporal dissociation of the target-congruency effects generated by different types of gazes. For example, using fMRI or MEG to examine the neural correlates of the target-congruency effects of convergent and non-convergent gazes, respectively, can tell whether the target-congruency effects of convergent gazes exists in both sensory cortex and frontoparietal cortices, while that of non-convergent gazes only becomes evident in frontoparietal cortices related to decisional processes.

Admittedly, there were additional differences between the convergent and non-convergent gaze condition, such as numerosity (2 gaze cues vs. 1 gaze cue) and motion (2 gaze shifts vs. 1 gaze shift), which may affect gaze cueing of attention. However, these factors do not seem to fully account for the present findings, particularly N170 and P3 results. For example, past research has showed that increased numerosity may augment N170^41^ or leave P3 unaffected^42^, and increased motion coherence may induce larger P3^43,44^, all of which were different from what we found in the current study. Therefore, instead of “low-level” factors of numerosity or coherence of motion, “high-level” factors such as perceived reliability may be a more reasonable explanation, in line with the possible evolutionary importance of majority’s gaze that indicates food locations or resources for survival^6^. Further evidence could be collected, for example, by manipulating the number of gaze shift in formulating convergent and non-convergent gazes, or the number of faces presenting different types of gazes, to scrutinize the exact role “low-level” factors play in such multiple gaze cueing processes. Neuroimaging techniques may also be useful to test whether “low-level” factors contribute to the gaze convergence effect, as possible effects by “low-level” factors on gaze cueing would be much more likely reflected in activities in sensory cortex related to perceptual processing rather than in frontoparietal cortex related to decisional processing or social cognition.

In conclusion, our study expanded existing research on the effect of gaze convergence on social attention, and explored the basic neural mechanism of the cueing effect from multiple social gazes. We found that when the gazes from two human avatars converged, they induced quicker reaction time and a larger target-congruency effect from the observer comparing to when they did not. Our exploration of the neural mechanism suggests that gaze convergence enhances the perceptual processing and the expectation of the congruent target. Our study used a highly simplified model of multi-person social interaction. Future studies may benefit from introducing variation in the temporal, spatial and social features of the interaction scenario to further investigate the cognitive mechanism underlying the perception of social gazes. Also, brain imaging techniques such as fMRI may help further investigate the perceptual and decisional impacts of shared gazes suggested by the present study.
